# *Yihx*-encoded haloacid dehalogenase-like phosphatase HAD4 from *Escherichia coli* is a specific α-d-glucose 1-phosphate hydrolase useful for substrate-selective sugar phosphate transformations

**DOI:** 10.1016/j.molcatb.2014.09.004

**Published:** 2014-12

**Authors:** Martin Pfeiffer, Patricia Wildberger, Bernd Nidetzky

**Affiliations:** a Institute of Biotechnology and Biochemical Engineering, Graz University of Technology, Petersgasse 12/1, A-8010 Graz, Austria; b ACIB – Austrian Centre of Industrial Biotechnology, Petersgasse 14, A-8010 Graz, Austria

**Keywords:** Phosphatase, HAD superfamily, Sugar phosphate, Substrate selectivity, Oriented immobilization

## Abstract

Phosphomonoester hydrolases (phosphatases; EC 3.1.3.) often exhibit extremely relaxed substrate specificity which limits their application to substrate-selective biotransformations. In search of a phosphatase catalyst specific for hydrolyzing α-d-glucose 1-phosphate (αGlc 1-*P*), we selected haloacid dehalogenase-like phosphatase 4 (HAD4) from *Escherichia coli* and obtained highly active recombinant enzyme through a fusion protein (Z_basic2__HAD4) that contained Z_basic2_, a strongly positively charged three α-helical bundle module, at its N-terminus. Highly pure Z_basic2__HAD4 was prepared directly from *E. coli* cell extract using capture and polishing combined in a single step of cation exchange chromatography. Kinetic studies showed Z_basic2__HAD4 to exhibit 565-fold preference for hydrolyzing αGlc 1-*P* (*k*_cat_/*K*_M_ = 1.87 ± 0.03 mM^−1^ s^−1^; 37 °C, pH 7.0) as compared to d-glucose 6-phosphate (Glc 6-*P*). Also among other sugar phosphates, αGlc 1-*P* was clearly preferred. Using different mixtures of αGlc 1-*P* and Glc 6-*P* (e.g. 180 mM each) as the substrate, Z_basic2__HAD4 could be used to selectively convert the αGlc 1-*P* present, leaving back all of the Glc 6-*P* for recovery. Z_basic2__HAD4 was immobilized conveniently using direct loading of *E. coli* cell extract on sulfonic acid group-containing porous carriers, yielding a recyclable heterogeneous biocatalyst that was nearly as effective as the soluble enzyme, probably because protein attachment to the anionic surface occurred in a preferred orientation via the cationic Z_basic2_ module. Selective removal of αGlc 1-*P* from sugar phosphate preparations could be an interesting application of Z_basic2__HAD4 for which readily available broad-spectrum phosphatases are unsuitable.

## Highlights

• Functional expression of *Escherichia coli* haloacid dehalogenase-like phosphatase 4 (HAD4).
• Characterization of HAD4 fusion to the cationic module Z_basic2_.
• Single-step capture and polishing purification of Z_basic2__HAD4.
• Selective conversion of α-d-glucose 1-phosphate in mixture with glucose 6-phosphate.
• Oriented immobilization with high effectiveness of Z_basic2__HAD4 on porous support.

## Graphical abstract

**Figure:**
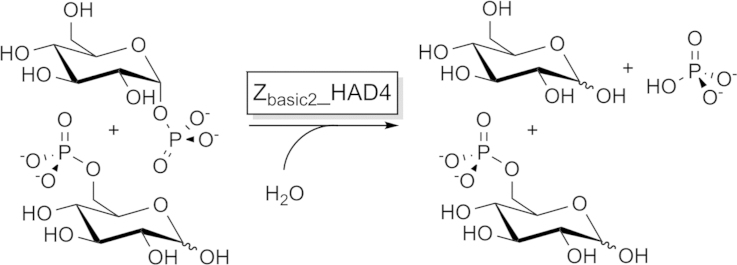

## 1 Introduction

Approximately half of the intracellular carbohydrates in the *Escherichia coli* metabolome contain a phosphate moiety [1]. A plethora of enzymes is involved in the dynamic regulation and homeostasis of sugar phosphate metabolite levels. One key enzyme subset is the phosphatases [2]. They catalyze phosphomonoester group hydrolysis to release inorganic phosphate from phosphorylated substrate.

The known sugar phosphate phosphatases represent a large diversity in protein structures, catalytic mechanisms, and substrate specificity. However, many of them are evolutionary related to haloacid dehalogenase (HAD)-like proteins [3], [4], [5]. Originally named after the activity of its first structurally characterized member (l-2-HAD from *Pseudomonas* sp. YL), the HAD-like protein superfamily currently comprises mainly phosphatases, with about 79% of the classified and biochemically studied proteins exhibiting this activity [6].

Common structural element of HAD-like phosphatases is a Rossmann fold-like α/β core domain that contains the active site. According to the presence and structure of an additional domain, the so-called CAP, HAD-like phosphatases are subdivided into subfamilies CI (α-helical CAP), CII (mixed α/β CAP), and CIII (no CAP) [7]. The catalytic centre is highly conserved. It features a key Asp that is proposed to function as catalytic nucleophile in a double displacement-like enzyme reaction mechanism. Phospho-monoester hydrolysis would accordingly proceed in two catalytic steps via a covalent aspartyl-phosphate enzyme intermediate [6], [7], [8]. HAD-like phosphatases require divalent metal ion (typically Mg^2+^) bound in the active site for their full activity [9], [10]. Phosphatase substrate specificity is dictated by flexible loop structures on the α/β core domain and on the CAP domain [11]. The spectrum of phosphoryl substrates hydrolyzed is usually very broad, and it includes both sugar and non-sugar phosphates [12]. Relationships between structure and specificity are only weakly defined for HAD-like phosphatases. Substrate binding and product release involve domain-closing and opening movements of the CAP [9], [13], [14], [15]. Protein conformational flexibility further complicates efforts to infer substrate specificity from sequence and three-dimensional structure information alone. Purposeful selection or molecular design of a HAD-like phosphatase for biocatalytic conversion of specific target substrates is therefore difficult. Evidence from biochemical characterization is vital for efficient development.

Selectivity is a prime feature of many enzymatic transformations [16]. A growing number of examples show that in a comparison of conventional-chemical and biocatalytic process options in organic synthesis [17], [18], it is usually the bio-based selectivity that provides a decisive advantage for process development. In this paper, we address the problem of substrate-selective hydrolysis of α-d-glucose 1-phosphate (αGlc 1-*P*) by phosphatases and report identification of HAD4 (*yihx* gene product) from *E. coli* for that purpose. Major demand for the reaction is in reactive processing of mixtures of sugar phosphates to eliminate all of the αGlc 1-*P* present in the starting material. Sample work-up for analytics, in the field of sugar phosphate metabolomics for example, and facilitated sugar phosphate product recovery constitute interesting applications of selective αGlc 1-*P* converting phosphatases. The readily available broad-spectrum phosphatases are however not suitable for these applications, and discovery of new phosphatase catalysts is therefore required. HAD4 was obtained as highly active recombinant enzyme through a specially designed fusion protein (Z_basic2__HAD4) that contained Z_basic2_, a strongly positively charged three α-helical bundle module, at its N-terminus [19]. Z_basic2_ facilitated functional expression of soluble protein in *E. coli* and enabled protein capture and polishing efficiently combined in a single step of cation exchange chromatography. Furthermore, the Z_basic2_ module had an instrumental role in the development of an immobilized HAD4 biocatalyst where Z_basic2__HAD4 was bound directly from crude bacterial cell extract on sulfonic acid group-containing porous carriers. Attachment of fusion protein to the negatively charged carrier surface was not only highly selective in that accompanying *E. coli* protein only showed marginal binding under the conditions used, but it also appeared to have occurred in a strongly preferred orientation via the cationic binding module: immobilized Z_basic2__HAD4 was nearly as effective as the free enzyme. Kinetic characterization of Z_basic2__HAD4 with αGlc 1-*P* and the competing sugar phosphate substrate d-glucose 6-phosphate (Glc 6-*P*) is reported. Utilization of different sugar phosphates as substrates for hydrolysis by Z_basic2__HAD4 is described, and some basic properties of the enzyme fused to Z_basic2_ are reported. Clear-cut separation of Glc 6-*P* from αGlc 1-*P* through selective hydrolysis of the sugar 1-phosphate at high concentration (up to 180 mM) and in the presence of up to 9-fold excess of competing substrate is shown.

## 2 Materials and methods

### 2.1 Chemicals and reagents

Unless stated otherwise, all chemicals were of highest purity available from Sigma–Aldrich (Vienna, Austria) or Roth (Karlsruhe, Germany). Sugar phosphates were obtained as sodium or ammonium salts. Oligonucleotide primers were from Sigma–Aldrich (Vienna, Austria). DNA sequencing was done at LGC Genomics (Berlin, Germany).

### 2.2 Expression and purification of His_6__HAD4

Expression vector pCA24N-yihx encoding HAD4 equipped with N-terminal hexahistidine tag was kindly provided by Dr. Alexander Yakunin. The vector was transformed into electro-competent cells of *E. coli* BL21 (DE3), and single-colony transformants were selected on agar plates containing 0.025 mg/mL chloramphenicol. Recombinant protein was produced in 1-L baffled shaking flasks at 37 °C using an agitation rate of 110 rpm (Certomat BS-1 shaking incubator, Sartorius, Göttingen, Germany). Flasks contained 250 mL Lennox-medium supplemented with 0.025 mg/mL chloramphenicol. At OD_600_ of 0.8, temperature was decreased to 18 °C, 0.01 mM isopropyl-β-d-thiogalactopyranoside (IPTG) was added, and cultivation was continued for 20 h. Centrifuged (4 °C, 30 min; 4,420 g) and washed cells were suspended in 10 mL of 50 mM Mes pH 7.0 buffer and disrupted by triple passage through French pressure cell at 150 bar (American Instruments, Silver Springs, USA). After removal of cell debris (4 °C, 30 min; 20,000 g) and filtration through a 1.2 μm cellulose-acetate syringe filter (Sartorius, Göttingen, Germany). The pre-treated cell lysate (350 mg; 8 mL) was loaded on a 14 mL copper-loaded affinity column (Chelating Sepharose Fast Flow; XK 16/20 column, GE Healthcare, Little Chalfont, U.K.), beforehand equilibrated with buffer A (50 mM Tris HCl, pH 7.0 containing 300 mM NaCl) and mounted on an BioLogic DuoFlow™ system (Biorad, Hercules, CA, USA). Differential elution was performed with buffer B (50 mM Tris HCl, pH 7.0 containing 300 mM NaCl and 400 mM imidazole) at 10 °C and a flow rate of 3 mL/min. The elution protocol comprised 5 steps, whereas concentration of buffer B was stepwise increased to 0, 10, 30, 60 and 100% in a volume of 100, 90, 60, 30 and 75 mL, respectively. All buffers were degassed and filtered using 0.45 μm cellulose-acetate or 0.2 μm polyamide filters. Protein elution was monitored spectrophotometrically at 280 nm, and collected fractions were assayed for protein concentration and phosphatase activity against *p*-nitrophenyl phosphate (pNPP; see Assays). Active fractions were pooled and buffer was exchanged to buffer A_2_ (50 mM KH_2_PO_4_-buffer, pH 7.0) with Amicon Ultra-15 Centrifugal Filter Units (Millipore, Billerica, USA). Pooled fractions were further purified using a 50 mL Fractogel EMD-DEAE column (XK 26/20, Merck, Darmstadt, Germany) mounted on a BioLogic DuoFlow™ system. Protein elution was performed with buffer B_2_ (50 mM KH_2_PO_4_, 1 M NaCl, pH 7.0) applying a five-step purification protocol at a constant flow rate of 5 mL/min: step 1, isocratic flow of 100% buffer A_2_ for 50 mL; step 2, linear increase to 15% buffer B_2_ in 40 mL; step 3, isocratic flow of 85% buffer A_2_ and 15% buffer B_2_ for 80 mL; step 4, linear increase to 100% buffer B_2_ in 180 mL; step 5, isocratic flow of 100% buffer B_2_ for 70 mL. Active fractions were pooled and buffer was exchanged to 50 mM Hepes pH 7.0 with Amicon Ultra-15 Centrifugal Filter Units. Protein purification was monitored by SDS PAGE and phosphatase activity measurements.

Alternatively, the pre-treated cell lysate (280 mg; 8 mL) was loaded on 1.6 cm × 2.5 cm; 5 mL HisTrap FF column (GE Healthcare), equilibrated with buffer C (50 mM MES, pH 7.4 containing 125 mM NaCl, 20 mM imidazole) and mounted on an ÄKTA prime plus (GE Healthcare) system. Protein was eluted using an imidazole gradient from 0 to 80% buffer D (50 mM MES, pH 7.4 containing 125 mM NaCl and 500 mM imidazole) at 10 °C and at a flow rate of 4 mL/min. At 20% buffer D, the linear gradient was halted for 2 column volumes to facilitate differential elution of bound protein. Active fractions were pooled and buffer was exchanged to 50 mM MES, pH 7.0. Protein purification was monitored by SDS PAGE and phosphatase activity measurements. Densitometry evaluation of the SDS PAGE was performed with the GelAnalyzer software (http://www.gelanalyzer.com/index.html).

### 2.3 Molecular cloning, expression and purification of Z_basic2__HAD4

The HAD4 gene was amplified from pCA24N-yihx by PCR using Phusion DNA polymerase (Thermo Scientific, Waltham, USA) and the following pair of oligonucleotide primers:

5′-GAAGCTCTGTTCCAGGGTCCGCTCTATATCTTTGATTTAG-3′, and 5′-CTTTGTTAGCAGCCGGATCTCTTAGCATAACACCTTCG-3′.

PCR consisted of a pre-heating step at 98 °C for 5 min and was followed by 30 reaction cycles of denaturation at 98 °C for 30 s, annealing at 70 °C for 30 s, and elongation at 72 °C for 1.5 min. The final extension step was carried out at 72 °C for 5 min. The resulting PCR product comprised 5′- and 3′-overhangs complementary to the target sequence of the pT7ZbQGKlenow destination vector [19]. Vector pT7ZbQGKlenow was amplified by PCR using Phusion DNA polymerase and the following pair of oligonucleotide primers:

5′-CGGACCCTGGAACAGAGC-3′, and 5′-GAGATCCGGCTGCTAACAAAG-3′.

PCR protocol was the same as above, except that elongation at 72 °C was extended to 3 min. Amplification products of the two PCRs were individually subjected to parental template digest by *Dpn*I and then recombined via Gibson assembly [20]. The resulting construct encodes a fusion protein where the small module Z_basic2_ is joined by a flexible linker (11 amino acids) to the N-terminus of HAD4. Sequenced plasmid vector encoding the Z_basic2__HAD4 fusion protein was transformed into electrocompetent cells of *E. coli* BL21-Gold (DE3), and single-colony transformants were selected on agar plates containing 0.05 mg/mL kanamycin.

Protein production was done as described above, except for the use of 0.05 mg/mL kanamycin. Harvested cells were resuspended in 10 mL of buffer A_3_ (50 mM MES buffer, pH 7.0, containing 100 mM NaCl). Preparation of cell extract was done exactly as already described.

Protein was purified at 10 °C using pre-packed 1.6 cm × 2.5 cm; 5 mL HiTrap SP FF column (Ni Speharose; 16/25 mm, GE Healthcare) on ÄKTA prime plus system. The column was equilibrated with buffer A_3_ and 8 mL of the pre-treated cell extract were loaded onto the column. Protein elution was performed using a continuous salt gradient ranging from 0 to 100% of buffer B_3_ (50 mM HEPES buffer supplemented with 2 M NaCl, pH 7.0) in 150 mL at a flow rate of 4 mL/min. The target protein eluted at 50% buffer B_3_. Pooled fractions were collected and buffer was exchanged to 50 mM MES, pH 7.0, containing 250 mM NaCl and 50 mM MgCl_2_, as described before. The concentrated preparation was aliquoted and stored at −20 °C. Protein purification was monitored by SDS PAGE and phosphatase activity measurements.

### 2.4 Assays

Protein was measured with Roti-Quant reagent (Roth, Karlsruhe, Germany) referenced against bovine serum albumin (BSA). Purified Z_basic2__HAD4 was measured by UV absorbance at 280 nm using a molar extinction coefficient of 25440 M^−1^ cm^−1^, calculated from the sequence with ProtParam analysis tool from the expasy server [21]. Standard phosphatase activity was measured with pNPP (4.0 mM), the release of p-nitrophenol (pNP) was recorded continuously at 405 nm with a Beckman Coulter DU 800 UV/VIS spectrophotometer (Krefeld, Germany).

Reaction was performed at 30 °C using 50 mM MES, pH 7.0, containing 25 mM MgCl_2_ and 100 mM NaCl. One unit of activity (U) is the amount of enzyme releasing 1 μmol of pNP under the conditions used. Specific activity is expressed in U/mg protein.

Inorganic phosphate was measured with a spectrophotometric assay described by Saheki et al. [22]. Shortly, free phosphate forms a complex with molybdate and the phosphomolybdate complex is reduced by l-ascorbic acid in the presence of Zn^2+^, which produces a chromophore that can be measured at 850 nm.

Reported coupled enzyme assays were used to measure αGlc 1-*P* and Glc 6-*P* [23]. In contrast to Eis and Nidetzky [23], however, no trehalose phosphorylase was present in our reaction mixture. Glucose 6-*P* dehydrogenase from *Leuconostoc mesenteroides* (G8404; Sigma Aldrich) and α-phosphoglucomutase from rabbit muscle (P3397; Sigma Aldrich) were added sequentially. After addition of glucose 6-*P* dehydrogenase or phosphoglucomutase, formation of NADH was monitored at 340 nm and the NADH end concentration was used to calculate the corresponding Glc 6-*P* or αGlc 1-*P* concentration.

### 2.5 Kinetic characterization

All reactions were performed in 50 mM MES buffer, pH 7.0, containing 25 mM MgCl_2_ and 100 mM NaCl. Incubations were done at 37 °C using agitation at 650 rpm on a Thermomixer comfort (Eppendorf, Hamburg, Germany). αGlc 1-*P* or Glc 6-*P* was used as substrate in the concentration range 0.1–30 mM and 0.1–700 mM, respectively. To account for the lower activity of Z_basic2__HAD4 with Glc 6-*P* as compared to αGlc 1-*P*, the corresponding enzyme concentration was 0.39 (αGlc 1-*P*) and 16 μM (Glc 6-*P*), respectively. Reactions were started by addition of enzyme and allowed to run for up to 120 min while taking samples every 15 min. Thermal inactivation (99 °C, 5 min) was used to stop reactions. Free phosphate concentration was measured in supernatants of centrifuged samples.

It was confirmed that phosphate release was linear with incubation time. Phosphate concentration after 15 min, or 120 min of reaction was used to determine the initial rate for αGlc 1-*P* and Glc 6-*P* conversions, respectively. Phosphate blanks from reactions lacking enzyme were recorded.

Kinetic parameters were obtained from non-linear least squares fits (SigmaPlot, Systat, Erkrath, Germany) of initial rates to Michaelis–Menten equation expanded to include substrate inhibition (1). *V* is the initial rate, *V*_max_ is the maximum initial rate, *K*_M_ is the Michaelis constant, *K*_iS_ is the substrate inhibition constant, and [S] is the initial substrate concentration. Under conditions where *K*_iS_ < *K*_M_ (see later under Results) fitting of Eq. (1) did not succeed and individual determination of all three kinetic parameters was not possible. However, *V*_max_/*K*_m_ could be determined from the initial part of the *V*–[S] curve where *V* is linearly dependent on [S].

(1) $$V=\frac{V_{\max}\text{S}}{K_{\text{M}}+\text{S}(1+(\text{S}/K_{\text{iS}}))}$$

Besides αGlc 1-*P* and Glc 6-*P* various other sugar phosphates were examined as substrates of Z_basic2__HAD4: *α*-d-galactose 1-*P* (αGal 1-*P*), *α*-d-mannose 1-*P* (αMan 1-*P*), *α*-d-galactose 6-*P* (αGal 6-*P*) and *α*-d-mannose 6-*P* (αMan 6-*P*). They were used at constant concentration of 20 mM, and the initial rate of phosphate release was measured.

### 2.6 pH and temperature profiles of activity and stability

pH effects were studied at 37 °C (activity) or 25 °C (stability) in the range 4.0–9.0 using multicomponent buffer of 50 mM sodium acetate, 50 mM TES, 50 mM MES supplemented with 100 mM NaCl and 25 mM MgCl_2_. Temperature effects (20–60 °C) were examined at pH 7.0 using the salt-supplemented 50 mM MES buffer. pH or temperature effects on activity were determined using αGlc 1-*P* (20 mM) as substrate and measuring the phosphate release within 10 min of incubation. Effects on stability were determined from non-agitated incubations of enzyme solution (200 μL; 23 μM). Samples were withdrawn at certain times and residual activity was measured under αGlc 1-*P* assay conditions (pH) as described above.

### 2.7 Z_basic2__HAD4 immobilization

Basic protocol from Wiesbauer et al. [24] was used. Briefly, Fractogel EMD SO_3_^−^ (Merck, Darmstadt, Germany) and Relisorb SP400 (Resindion, Binasco Milano, Italy) were the carriers used, and their technical specifications are provided in Supplementary Table T1. Hundred mg of wet carrier were weighed into Eppendorf tubes, washed twice with water, and equilibrated with 50 mM MES (pH 7.0) containing 200 mM NaCl. Two mL of enzyme solution (5 mg/mL *E. coli* cell extract; 0.5 mg/mL purified Z_basic2__HAD4) were added and the tubes were incubated in an end-over-end rotator at 25 °C for 60 min. Supernatant was removed (25 °C, 5 min; 20,000 g) and weakly bound protein was washed away twice with loading buffer. Note that only at 2 M NaCl the specifically and strongly bound protein was eluted from the carrier. Enzyme immobilizates were stored in 50 mM MES (pH 7.0) containing 100 mM NaCl and 25 mM MgCl_2_ at 4 °C. Enzyme immobilization was monitored from the decrease in protein concentration and phosphatase activity in solution, observing that enzyme was stable during the immobilization.

Effectiveness factors (*η*) of the Z_basic2__HAD4 immobilizates were determined by comparing the observable activity of the carrier-bound enzyme to the theoretically bound enzyme activity calculated from the balance in solution. Therefore, *η* is the ratio of observable and theoretically bound enzyme activity. The relatively high phosphatase background in the *E. coli* cell extract restricted *η* determination to conditions where purified Z_basic2__HAD4 was immobilized. To measure the activity of the immobilized enzyme, 3 mg of immobilizate were incubated at 37 °C in 500 μL solution containing 20 mM αGlc 1-*P* as substrate. Agitation was at 330 rpm to keep particles in suspension. Release of phosphate was measured in samples after 10 min of incubation.

### 2.8 Substrate-selective hydrolysis of αGlc 1-P in the presence of Glc 6-P using free or immobilized Z_basic2__HAD4

Reaction conditions were the same as above under *2.5 Kinetic characterization* except that temperature was 30 °C unless indicated. The substrate and soluble enzyme concentrations used are shown under *3. Results*. Samples were taken at certain times and assayed for αGlc 1-*P* and Glc 6-*P*. Immobilized enzyme prepared from *E. coli* cell extract was used identically as the free enzyme, applying 3 mg of carrier in 500 μL substrate solution. Agitation at 330 rpm with a magnetic stirrer bar was sufficient to have carrier particles well suspended. Once αGlc 1-*P* was converted almost exhaustively (∼90 min), particles were spinned down (25 °C, 5 min; 20,000 g), washed once with buffer, and then suspended again with substrate solution. Restart of the reaction was done twice.

## 3 Results and discussion

### 3.1 Functional expression and purification of HAD4

Kuznetsova et al. reported isolation of μg amounts of His_6__HAD4 using automated high-throughput work-up procedures performed at μL scale [12]. We attempted to scale up the purification of His_6__HAD4 to a processing capacity of about 400 mg total protein from the crude *E. coli* cell extract. Contrary to the earlier study where Ni-NTA beads were used [12], Cu^2+^ loaded immobilized metal affinity chromatography (IMAC) in 14 mL column format (16 cm × 20 cm) was used herein. Even though about one-third of the applied protein was bound to the column, differential elution with an imidazole step gradient did not result in fractionation of protein(s) exhibiting clear phosphatase activity towards pNPP substrate. Protein pools potentially containing phosphatase activity showed a prominent band of correct size (25 kDa) next to other major contaminants in the SDS polyacrylamide gel (Supplementary Fig. S1). They were therefore further fractionated by anion exchange chromatography. Collected fractions showed only trace amounts of phosphatase activity, and their analysis by SDS PAGE indicated that purification had not been successful (Supplementary Fig. S1).

We therefore tested IMAC on Ni^2+^ Sepharose using a different column format (1.6 cm × 2.5 cm; 5 mL; HisTrapFF) at smaller scale. Pooled protein fractions exhibiting phosphatase activity were shown by SDS PAGE to contain the expected band of 25 kDa but also a major contaminant of around 37 kDa size (Supplementary Fig. S2). According to densitometric analysis of the protein gel, the sample was composed roughly of 30–40% His_6__HAD4. Yield of target protein was therefore estimated to have been around 5 mg/L culture.

To establish a more efficient procedure of isolation of HAD4 and also in view of convenient immobilization of the enzyme of ion-exchange support, we chose not to optimize protocols for His_6__HAD4, but opted for an alternative fusion protein approach. A chimeric enzyme termed Z_basic2__HAD4 was constructed where the so-called Z_basic2_ module was attached to the N-terminus of HAD4. Z_basic2_ is an engineered arginine-rich variant of the Z domain, a 58 amino acid (7 kDa) three-helix bundle obtained from the B domain of staphylococcal protein A [25]. Fusion to Z_basic2_ was known from previous studies to enhance solubility of proteins otherwise poorly soluble under conditions of recombinant production in *E. coli* [19], [26]. Z_basic2_ was furthermore used as affinity module for protein purification by cation exchange chromatography [25]. Finally, it was also applied for non-covalent immobilization of different enzymes on negatively charged carrier surfaces [24], [27].

**Fig. 1:**
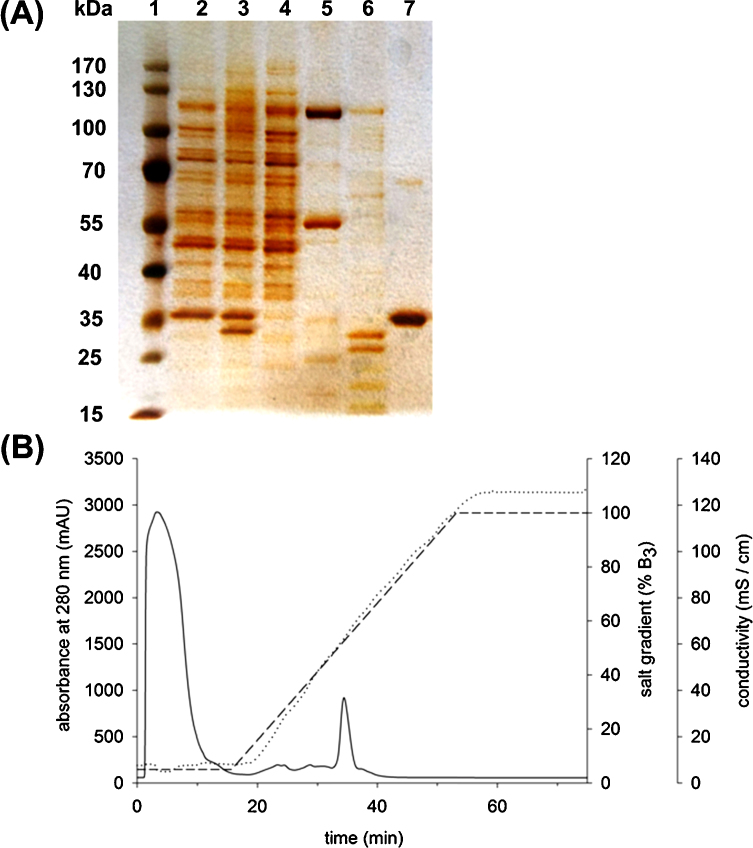
(A) Purification of Z_basic2__HAD4 monitored by SDS-PAGE. 1: PAGE-ruler protein ladder, 2: cell extract (1.5 μg), 3: insoluble protein fraction from *E. coli* expression culture (1.5 μg), 4: flow-through (proteins that did not bind to the column) (1.5 μg), 5: fraction with minor phosphatase activity (1.0 μg), 6: fraction with minor phosphatase activity (0.5 μg), 7: purified Z_basic2__HAD4 (1.5 μg); (B) Chromatogram of Z_basic2__HAD4 purification with 1.6 cm × 2.5 cm; 5 mL HiTrap SP FF column. The absorbance trace at 280 nm is shown as solid line. The salt gradient used is shown as dashed line, and the measured conductivity is shown as dotted line.

Z_basic2__HAD4 was isolated by cation exchange chromatography of the *E. coli* cell extract on HiTrap SP FF resin. Purified protein exhibited the expected size of 31 kDa. Capture of target protein, from the complex protein matrix and its purification to apparent homogeneity (Fig. 1A) were combined efficiently in a single work-up step. Z_basic2__HAD4 eluted from the column as discrete protein peak at high salt concentration, as shown in Fig. 1B. Analysis by SDS PAGE (Fig. 1A) revealed that the flow-through fraction did not contain Z_basic2__HAD4, demonstrating that all of the applied target protein had bound to the column. Fig. 1A also shows that insoluble protein recovered from the *E. coli* cells contained significant amounts of Z_basic2__HAD4, indicating that fusion to Z_basic2_ was not fully sufficient to prevent aggregation of HAD4 during recombinant production in *E. coli*. About 11 mg of pure Z_basic2__HAD4 were recovered from each L of bacterial cell culture. Purification with HiTrap SP FF resin was extremely effective, considering that the total protein amount present in the sample was almost 270 mg/L. It is interesting but currently not understood why the purification of Z_basic2__HAD4 was much more efficient than purification of His_6__HAD4. The specific activity towards pNPP of Z_basic2__HAD4 was determined as 0.06 U/mg.

Stock solutions of purified Z_basic2__HAD4 were prepared by concentrating desalted protein in 50 mM MES buffer, pH 7.0, to about 10 mg/mL. After frozen storage at −20 °C, we observed significant protein precipitation in thawed samples. Addition of 250 mM NaCl resulted in partial re-solubilization of the precipitate concomitant with recovery of some of the original phosphatase activity in solution, suggesting effect of ionic strength on solubility and stability of Z_basic2__HAD4. Supplementing the storage buffer with a combination of salts (250 mM NaCl, 50 mM MgCl_2_) prevented the precipitation of Z_basic2__HAD4 completely. Storage in liquid state at 4 °C was preferred where the enzyme was stable for several weeks. Tentative explanation for tendency of Z_basic2__HAD4 to form insoluble aggregates at low ionic strength is the large difference in the calculated isoelectric points for Z_basic2_ (*pI* = 11.5) and HAD4 (*pI* = 5.18). Intramolecular and intermolecular domain–domain interactions driven by charge complementarity might promote a native-like protein aggregation that is partly reversible at high salt concentration. Mg^2+^ ions could have two effects in this context. First of all, Mg^2+^ is an active-site ligand of HAD4 [12]. Thus the structure of the catalytic center (and perhaps that of the whole protein) might be stabilized when Mg^2+^ is bound. Secondly, adsorption of Z_basic2_ fusions to negatively charged surfaces is strongly weakened in the presence of Mg^2+^ ions [28]. Capacity of Mg^2+^ to perturb charged interactions of the Z_basic2_ module are much more pronounced than that of Na^+^ (each as chloride salt) [28].

### 3.2 Kinetic analysis of hydrolysis of αGlc 1-P, Glc 6-P and other sugar phosphates catalyzed by Z_basic2__HAD4

Initial reaction rates at steady state were obtained from time-course measurements of substrate consumption and phosphate release. Disparity of initial rates determined from the different measurements would have indicated a phosphoryl transfer side reaction occurring next to the hydrolysis. Both the phosphorylated substrate and the glucose product could in principle function as acceptor of the phosphoryl group and thereby compete with water in the overall reaction. However, it was confirmed that initial rates of *substrate utilization* and *substrate hydrolysis* were identical within limits of experimental error.

**Fig. 2:**
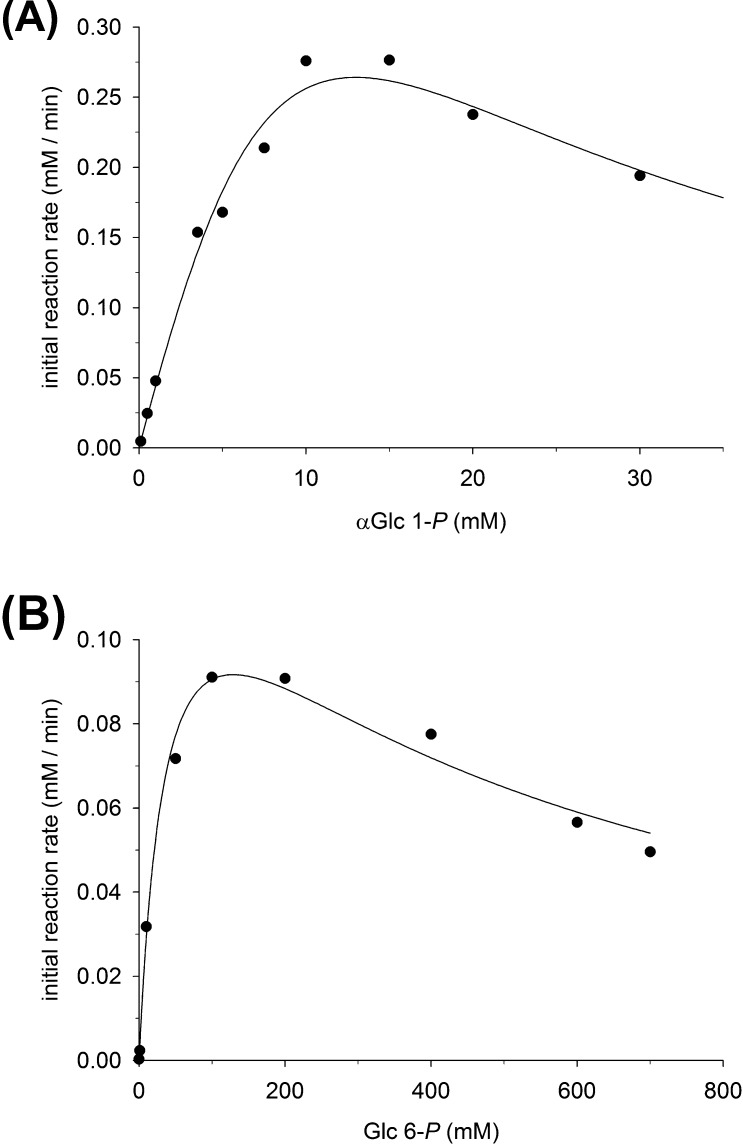
Initial-rate kinetic characterization of sugar phosphate conversion by Z_basic2__HAD4. (A) Michaelis–Menten plot for the hydrolysis of αGlc 1-*P.* (B) Michaelis-Menten plot for the hydrolysis of Glc 6-*P*. For further details about methodology, see *2.5. Kinetic characterization*. Symbols show the data, and the solid lines are non-linear fits of Eq. (1) to the data.

Fig. 2 shows initial rates determined at different substrate concentrations for hydrolysis of αGlc 1-*P* (Fig. 2A) and Glc 6-*P* (Fig. 2 B). For each substrate, the *V*–[S] curve increased at low [S] to reach a distinct maximum value of *V* at intermediate [S], only to decrease again in the high-[S] range. The kinetic behavior indicates inhibition of Z_basic2__HAD4 by the phosphorylated substrate. Interestingly, His_6__HAD4 did not show substrate inhibition at high αGlc 1-*P* (data not shown), suggesting substrate inhibition to be a feature caused by introduction of the Z_basic2_ module.

αGlc 1-*P* differed from Glc 6-*P* in affinity and reactivity for Z_basic2__HAD4-catalyzed hydrolysis. Maximum value of *V* was observed at around 10 mM for αGlc 1-*P* while for Glc 6-*P* the corresponding substrate concentration was about 100 mM (Fig. 2). Substrate inhibition also occurred at lower concentrations of αGlc 1-*P* as compared to Glc 6-*P*. An apparent *k*_cat_ was calculated from the maximum *V* value (*k*_cat_app_ = *V*_max_app_/[E]), and αGlc 1-*P* (10.2 s^−1^) was about 110-fold more active in *k*_cat_app_ terms than Glc 6-*P* (0.09 s^−1^). By way of comparison, *k*_cat_app_ for hydrolysis of pNPP (4 mM) was 0.032 s^−1^. For comparison with Z_basic2__HAD4, we also examined the preparation of His_6__HAD4 under otherwise identical conditions and measured its hydrolytic activity against αGlc 1-*P* (10 mM). Using correction for partial purity of the enzyme preparation used where only 30–40% of total protein was His_6__HAD4 (Fig. S2), we calculated a *k*_cat_app_ between 7.1 and 9.5 s^−1^ from the experimentally determined initial rate. Therefore, this result is important in showing that fusion to Z_basic2_ did not diminish the intrinsic activity of HAD4 relative to the His_6__HAD4 reference.

Eq. (1) could be used for data fitting by nonlinear least squares regression, as shown in Fig. 2; however, the resulting kinetic parameters (*k*_cat_, *K*_M_, *K*_iS_) were not well defined statistically. Covariance analysis revealed strong interdependence of all parameters, but especially of *K*_M_ and *K*_iS_, implying that independent determination of each single parameter was not possible from the data available. An approximate ratio between *K*_M_/*K*_iS_ can be given however, and this was ∼21 (60/2.82) for αGlc 1-*P* and ∼0.15 (51/351) for Glc 6-*P*. To nevertheless obtain parameter of enzyme specificity, we determined *k*_cat_/*K*_M_ from the part of the *V*–[S] curve where *V* was linearly dependent on [S]. The relationship *V* = (*k*_cat_/*K*_M_) [S] [E] holds under these conditions. *k*_cat_/*K*_M_ for hydrolysis of αGlc 1-*P* (1.87 ± 0.03 mM^−1^ s^−1^) exceeded the *k*_cat_/*K*_M_ for hydrolysis of Glc 6-*P* (3.2 ± 0.1 × 10^−3^ mM^−1^ s^−1^) by 565-fold. Z_basic2__HAD4 strongly discriminates between αGlc 1-*P* and Glc 6-*P* as substrate for hydrolysis.

**Fig. 3:**
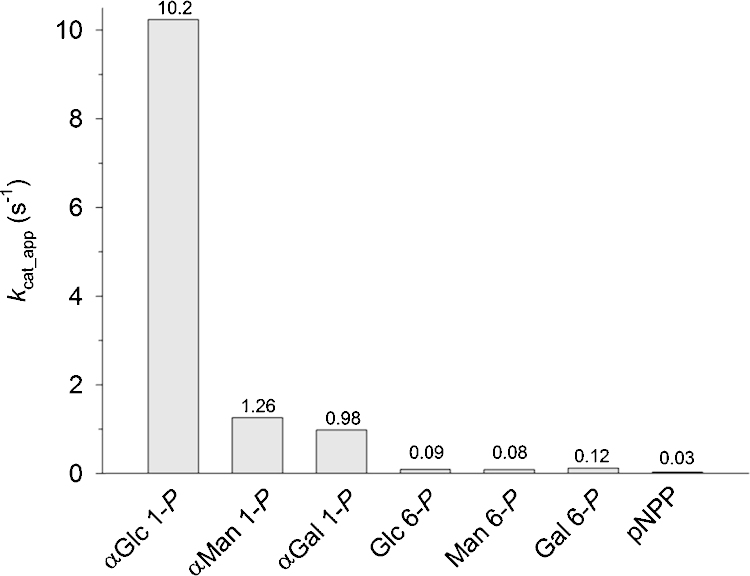
Phosphatase activity of Z_basic2__HAD4 with different sugar phosphate substrates and pNPP. Reactions were performed at 37 °C in 50 mM MES buffer (pH 7.0) containing 25 mM MgCl_2_ and 100 mM NaCl. The substrate concentration was 20 mM, except for pNPP (4.0 mM). *k*_cat_app_ is an apparent turnover number calculated from the initial rate of phosphate release divided by the molar enzyme concentration which in turn is obtained from the protein concentration and the enzyme molecular mass of 31 kDa. For further details about methodology, see Section 2.5.

Besides αGlc 1-*P* and Glc 6-*P* we examined other sugar phosphates as substrates of Z_basic2__HAD4, focusing in particular on the enzyme's ability to discriminate between the α-configured 1-phosphate and the corresponding 6-phosphate. Fig. 3 shows the results. All sugar 6-phosphates (d-Glc, d-Man, d-Gal) were slow enzyme substrates, exhibiting turnover rates of about 1 × 10^−1^ s^−1^. Activity with αMan 1-*P* and αGal 1-*P* was enhanced by about one magnitude order in comparison (Fig. 3), yet the activity with αGlc 1-*P* stood out among all substrates tested. Z_basic2__HAD4 can be considered a relatively specific αGlc 1-*P* hydrolase, overall consistent with findings of Kuznetsova et al. on the substrate specificity of His_6__HAD4 [21].

### 3.3 Effects of pH and temperature on activity and stability of Z_basic2__HAD4

Results are shown in Figs. S3 (pH effects) and S4 (temperature effects). The pH-profile of Z_basic2__HAD4 activity at 37 °C was bell-shaped with a broad plateau between pH 5.0 and pH 7.0 where enzyme showed at least 85% of its maximum activity at pH 6.0. pH dependence of the activity is similar for Z_basic2__HAD4 and His_6__HAD4 [21]. Recorded at 25 °C, the enzyme was fully stable for up to 20 h at all pH values in the range 4.0–9.0. Temperature profile at pH 7.0 showed optimum activity of Z_basic2__HAD4 at 40 °C, with rapid decline of activity at higher temperatures. Activity was fully stable for 20 h at or below 30 °C while at the higher temperatures (≥ 40 °C) the activity was very rapidly lost.

### 3.4 Substrate-selective hydrolysis of αGlc 1-P in a mixture of αGlc 1-P and Glc 6-P

**Fig. 4:**
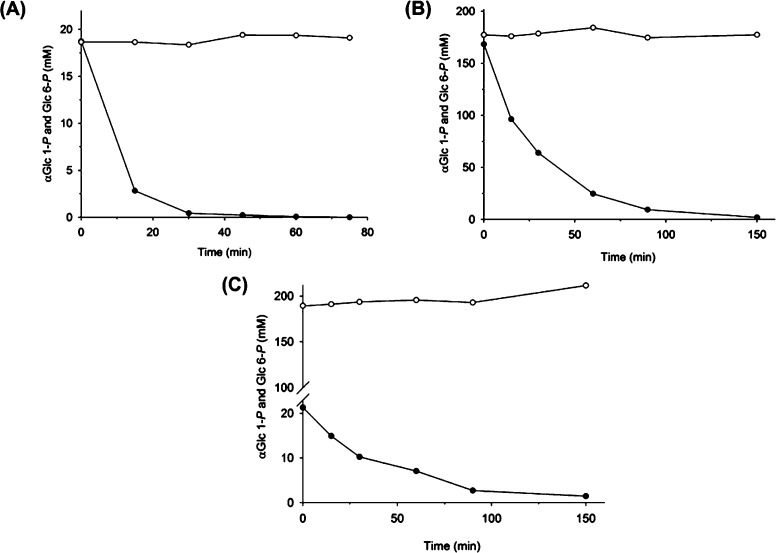
Selective hydrolysis of αGlc 1-*P* by Z_basic2__HAD4. Reaction mixtures contained (A) 20 mM αGlc 1-*P* (•) and 20 mM Glc 6-*P* (○), (B) 180 mM αGlc 1-*P* (•) and 180 mM Glc 6-*P* (○), and (C) 20 mM αGlc 1-*P* (•) and 180 mM Glc 6-*P* (○). Reactions further contained purified Z_basic2__HAD4 at 3.2 μM (A), 16 μM (B) and 1.6 μM (C). They were performed at 30 °C and pH 7.0, except in A where 37 °C was used. Note: the Glc 6-*P* concentration in panel C at 150 min was measured but includes effect of slight water evaporation.

High selectivity of Z_basic2__HAD4 for hydrolyzing αGlc 1-*P* as compared to Glc 6-*P*, as implied by the ratio of the corresponding *k*_cat_/*K*_M_ values, was put to critical test in a biotransformation experiment in which both sugar phosphates were offered as substrate. The substrate concentrations (20 mM; 180 mM) and also the molar ratio of αGlc 1-*P*/Glc 6-*P* were varied. Time courses of the enzymatic conversions are shown in Fig. 4 (panels A–C). In all cases, αGlc 1-*P* was effectively depleted from the substrate mixture while Glc 6-*P* was not utilized within error limit.

Z_basic2__HAD4-catalyzed selective hydrolysis of αGlc 1-*P* thus provided effective mean for separation of the two sugar phosphates. Specialized separation procedures such as borate complex ion exchange chromatography [29] would otherwise be needed to isolate the Glc 6-*P*. In the case of the alternative separation problem to remove Glc 6-*P* from αGlc 1-*P*, another HAD-like phosphatase, namely the enzyme BT4131 from *Bacteroides thetaiotaomicron*, could be of interest [9]. The phosphatase is highly active in hydrolysis of hexose 6-phosphates and pentose 5-phosphates, but exhibits barely any activity with hexose 1-phosphates. Taken together, these results suggest that HAD-like phosphatases present a potentially valuable source of selective sugar phosphate hydrolases that could be applied to resolution of mixtures of sugar phosphates that are otherwise very difficult to separate. In addition to phosphatases that exhibit broad substrate spectrum and are already available commercially, development of a toolbox of selective phosphomonoester hydrolases could provide interesting opportunities for biocatalysis.

### 3.5 Immobilization of Z_basic2__HAD4 and repeated use of immobilizates

Immobilization on Fractogel and Relisorb carriers was first done with the purified enzyme that was offered in a protein/wet carrier mass ratio of 0.01. Nearly all (≥95%) of the offered protein and phosphatase activity was bound to each carrier under the conditions used. An effectiveness factor (*η*) of 0.9 (±0.1) was determined for both immobilizates, indicating that immobilized Z_basic2__HAD4 was nearly as active as the corresponding soluble enzyme preparation. It was pointed out in earlier studies of Z_basic2__enzyme fusions [20], [23] and is confirmed here using Z_basic2__HAD4, that highly directed (“oriented”) immobilization via the Z_basic2_ module offers the particular advantage of preparing carrier-bound insoluble biocatalyst with optimized values of *η*. Note also that despite its noncovalent character, the immobilization is quite stable [24], [27], as will be shown later for Z_basic2__HAD4 immobilizates in biocatalyst recycling experiment.

When Fractogel or Relisorb carrier was charged with *E. coli* cell extract at protein/carrier mass ratio of 0.1, about 12–18% of total protein was bound to the solid support. Due to high background phosphatase activity in cell extract, balance for Z_basic2__HAD4 activity was not possible under these conditions. However, from the phosphatase activity of the immobilizate and the protein eluted from the carrier at high salt concentration, we determined a specific activity of immobilized Z_basic2__HAD4 of about 0.05 U/mg which is close to the specific activity of the purified soluble enzyme.

**Fig. 5:**
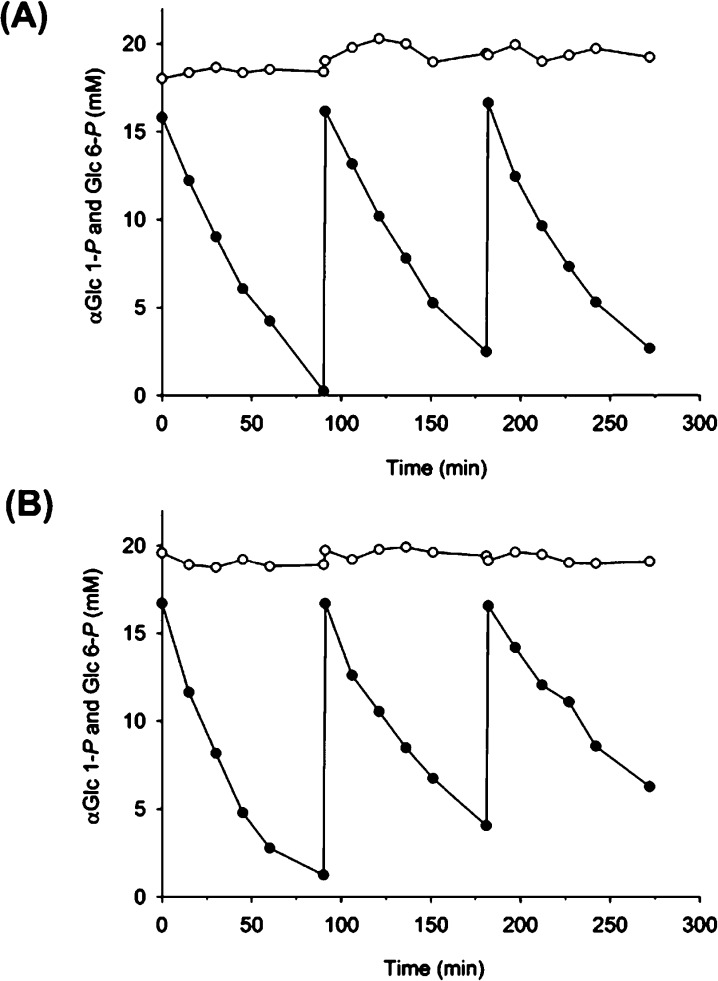
Selective hydrolysis of αGlc 1-*P* by Z_basic2__HAD4 immobilized on (A) Relisorb SP400 and (B) Fractogel EMD SO_3_^−^. Reaction mixtures contained 15 mM αGlc 1-*P* (•) and 20 mM Glc 6-*P* (○). Reactions were performed at 30 °C and pH 7.0. Three mg of immobilizate prepared from *E. coli* cell extract as described under *2.7. Z_basic2__HAD4 immobilization* were suspended in 500 μL substrate solution. Agitation was applied to keep particles in suspension.

Enzyme immobilizates thus prepared were applied to repeated batchwise hydrolysis of αGlc 1-*P* from a substrate mixture that contained 20 mM Glc 6-*P* and 15 mM of αGlc 1-*P*. Heterogeneous biocatalyst was recycled two times in each experiment, as shown in Fig. 5 where panels A and B display results for the Relisorb and Fractogel immobilizate, respectively. The Relisorb immobilizate was re-used without significant loss of activity over three reaction cycles where αGlc 1-*P* was degraded almost completely and the Glc 6-*P* present was not affected. Using the Fractogel immobilizate, by contrast, the rate of conversion of αGlc 1-*P* decreased about twofold from cycle 1 to 3 (Fig. 5, panel B). Assuming that Z_basic2__HAD4 was stable under the conditions used (cf. Fig. S4), gradual elution of enzyme from this carrier is a plausible reason for the observed decay of the reaction rate and thus the space-time yield. However, further examination of the operational stability of the Fractogel immobilizate of Z_basic2__HAD4 was beyond the scope of this study.

## 4 Conclusions

First-time functional expression, isolation, and characterization of *E. coli* HAD4 as fusion protein harboring the charged module Z_basic2_ at the N-terminus is reported. Single-step capture and polishing of Z_basic2__HAD4 by cation exchange chromatography was notably efficient to obtain multi-mg amounts of highly purified enzyme. Kinetic evidence and results from mixed sugar phosphate conversion experiments reveal that Z_basic2__HAD4 is highly selective for hydrolysis of αGlc 1-*P*. Z_basic2__HAD4 is proposed to be a useful catalyst for hydrolytic transformation (e.g. removal) of αGlc 1-*P* from complex substrate solutions containing multiple sugar phosphates. Immobilization of Z_basic2__HAD4 on porous carriers containing surface sulfonate groups provides a recyclable and highly effective heterogeneous biocatalyst. Z_basic2__HAD4 complements the current portfolio of phosphatases for use in biocatalysis by adding new characteristics of specificity and selectivity.
