# Supplementary material for: Yihx-encoded haloacid dehalogenase-like phosphatase HAD4 from Escherichia coli is a specific α-d-glucose 1-phosphate hydrolase useful for substrate-selective sugar phosphate transformations
Source: J Mol Catal B Enzym. 2014 Dec;110:39–46. doi: 10.1016/j.molcatb.2014.09.004 (PMC4251788; doi:10.1016/j.molcatb.2014.09.004)
Supplement: Supplementary file 1 [file mmc1.docx]

**SUPPLEMENTARY INFORMATION**


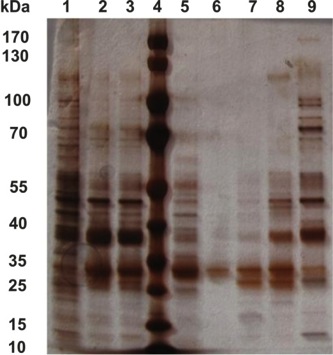


**Supplementary Figure S1.** His_6__HAD4 purification by Cu^2+^-IMAC and anion exchange chromatography monitored by SDS-PAGE. 1: Filtered *E. coli* crude extract loaded on the Cu^2+^-chelate column (2 µg), 2-3: Fractions after Cu^2+^-chelate column purification that contained phosphatase activity and were subsequently loaded on the DEAE-anion exchange column (2 µg each), 4: Page-Ruler protein ladder, 5: Flow-through (proteins that did not bind to the DEAE-anion exchange column) (1 µg), 6-9: Fractions of individual peaks which eluted at different concentrations of elution buffer during purification using the DEAE-anion exchange column (0.2 – 1.0 µg). Expected size of His_6__HAD4: 25 kDa.


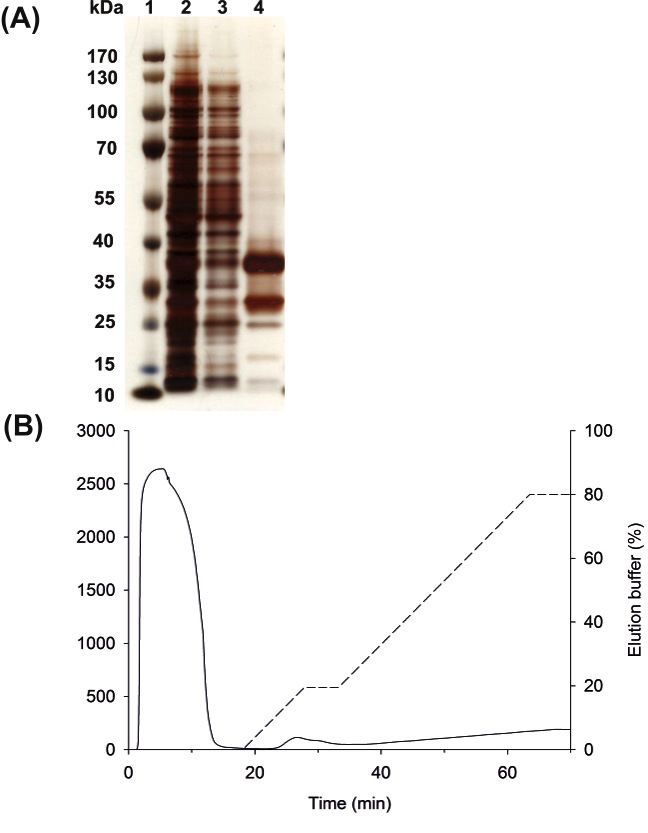


**Supplementary Figure S2. (A)** Purification of His_6__HAD4 by Ni^2+^-IMAC monitored by SDS-PAGE. 1: PAGE-ruler protein ladder, 2: Cell extract (2 µg), 3: Flow-through (proteins that did not bind to the column) (1.5 µg), 4: Purified His_6__HAD4 (1.5 µg); Expected size of His_6_-HAD4: 25 kDa. (**B)** Chromatogram of His_6__HAD4 purification performed with 2 × 5 mL HisTrap FF column. The absorbance trace at 280 nm is shown as solid line. The salt gradient used is shown as dashed line.


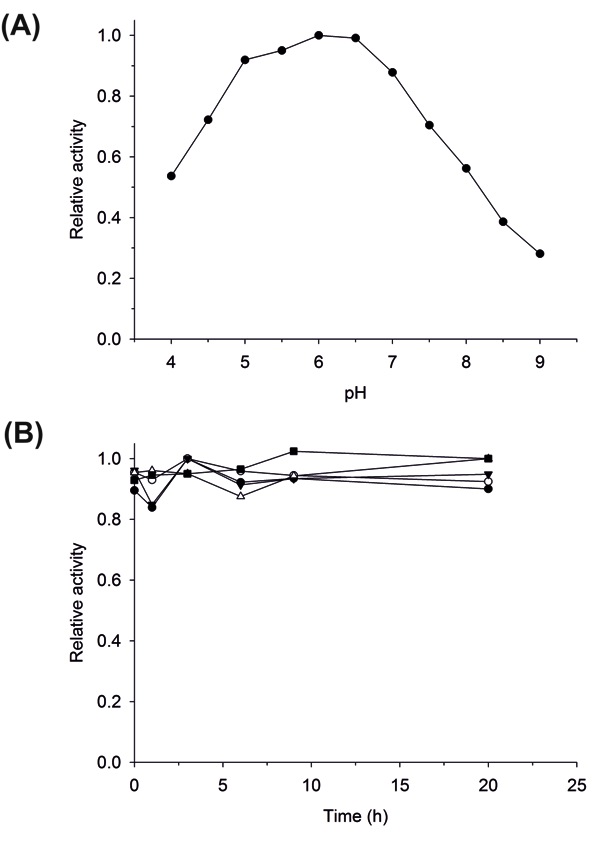


**Supplementary Figure S3. (A)** pH profile of Z_basic2__HAD4 phosphatase activity against αGlc 1-*P*. All reactions were performed in buffer mixture of 50 mM sodium acetate, 50 mM TES, and 50 mM MES supplemented with 100 mM NaCl and 25 mM MgCl_2_. Assays were performed at 37 °C using 20 mM αGlc 1-*P*. The concentration of purified enzyme was 1.5 µM. **(B)** pH effect on the stability of Z_basic2__HAD4 at 25 °C. Enzyme (1.5 µM) was incubated in the buffer described under (A) at pH 5.0 (full squares), pH 6.0 (open triangles), pH 7.0 (open circles), and pH 8.0 (full triangles). Enzyme activity remaining at times indicated was measured with the standard assay for αGlc 1-*P* hydrolysis.


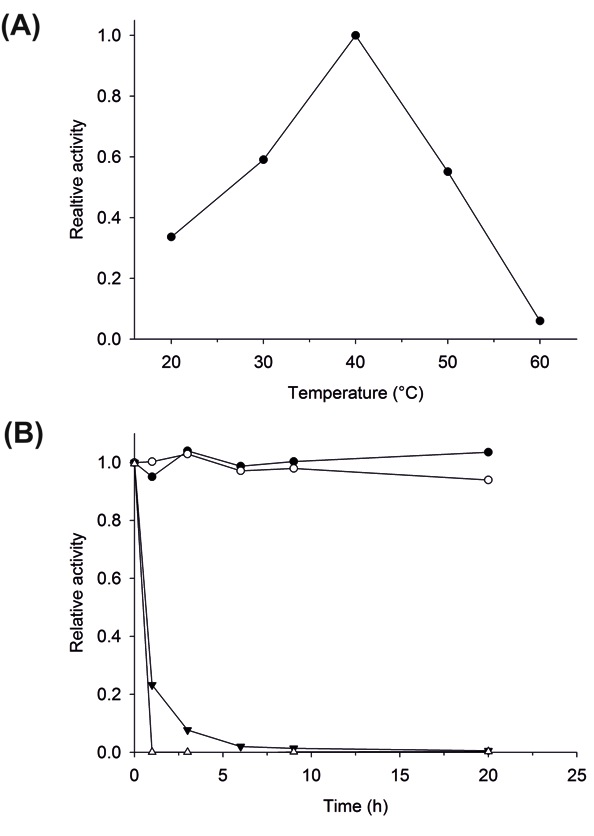


**Supplementary Figure S4.** Temperature profiles of activity **(A)** and stability **(B)** of Z_basic2__HAD4. All reactions were performed in 50 mM MES (pH 7.0) containing 100 mM NaCl and 25 mM MgCl_2_. The enzyme concentration used was 1.5 µM. In panel A, activity against 20 mM αGlc 1-*P* was measured. In panel B, activity remaining at the times indicated was measured with the standard assay for αGlc 1-*P* hydrolysis. Samples were incubated at 20°C (full circles), 30°C (open circles), 40°C (closed triangle), 50°C (open triangle).

**Supplementary Table T1.** Technical specifications of the carriers used.

| **Commercial product** | **Functional group** | **Particle size (µm)** | **Pore size (nm)** | **Dry matter^a^ (%)** | **Carrier form** |
| --- | --- | --- | --- | --- | --- |
| Fractogel EMD SO_3_^-^ | Sulfoisobutyl | 40-90 | 80 | 15 | Suspension |
| Relisorb SP400 | Sulfopropyl | 75-200 | 60-80 | 23 | Wet particles |

^a^) dry matter was determined after drying at 99°C for 3 hours.
